# Interaction of severe acute respiratory syndrome-coronavirus and NL63 coronavirus spike proteins with angiotensin converting enzyme-2

**DOI:** 10.1099/vir.0.2008/003962-0

**Published:** 2008-11

**Authors:** Alison C. Mathewson, Alexandra Bishop, Yongxiu Yao, Fred Kemp, Junyuan Ren, Hongying Chen, Xiaodong Xu, Ben Berkhout, Lia van der Hoek, Ian M. Jones

**Affiliations:** 1 School of Biological Sciences, University of Reading, Reading RG6 6AJ, UK; 2 Laboratory of Experimental Virology, Department of Medical Microbiology, Center for Infection and Immunity Amsterdam (CINIMA), Academic Medical Center of the University of Amsterdam, K3-110, Meibergdreef 15, 1105 AZ Amsterdam, The Netherlands

## Abstract

Although in different groups, the coronaviruses severe acute respiratory syndrome-coronavirus (SARS-CoV) and NL63 use the same receptor, angiotensin converting enzyme (ACE)-2, for entry into the host cell. Despite this common receptor, the consequence of entry is very different; severe respiratory distress in the case of SARS-CoV but frequently only a mild respiratory infection for NL63. Using a wholly recombinant system, we have investigated the ability of each virus receptor-binding protein, spike or S protein, to bind to ACE-2 in solution and on the cell surface. In both assays, we find that the NL63 S protein has a weaker interaction with ACE-2 than the SARS-CoV S protein, particularly in solution binding, but the residues required for contact are similar. We also confirm that the ACE-2-binding site of NL63 S lies between residues 190 and 739. A lower-affinity interaction with ACE-2 might partly explain the different pathological consequences of infection by SARS-CoV and NL63.

Severe acute respiratory syndrome (SARS) and NL63 coronaviruses (CoV) are two new members of the family *Coronaviridae* that have received considerable attention since their recent discovery (Drosten *et al.*, 2003; Kuiken *et al.*, 2003; Li *et al.*, 2003; Pyrc *et al.*, 2007; Sloots *et al.*, 2008; van der Hoek *et al.*, 2004). Infection by SARS-CoV causes severe acute respiratory syndrome with high levels of morbidity and mortality (Drosten *et al.*, 2003; Feng & Gao, 2007; Kuiken *et al.*, 2003), while infection by NL63 rarely requires hospitalization despite being commonly associated with lower respiratory tract infection and croup (van der Hoek, 2007; van der Hoek *et al.*, 2005; Wu *et al.*, 2008). Both viruses use angiotensin converting enzyme (ACE)-2 as the viral receptor (Hofmann *et al.*, 2005; Li *et al.*, 2003), raising the interesting possibility that the difference in pathology is (in part) the result of differential receptor engagement. Virus-mediated downregulation of ACE-2 has been suggested to underlie the pathology of SARS-CoV infection (Imai *et al.*, 2005; Kuba *et al.*, 2005) and it is unclear why this should not also be the case for NL63. Following engagement with ACE-2, the cellular pathways of internalization of the two viruses also appear to be different with SARS-CoV requiring the presence of the lysosomal cysteine protease cathepsin L to infect susceptible cells, while NL63 has no such requirement (Huang *et al.*, 2006). These data suggest that although both viruses utilize ACE-2 as the receptor, the consequences of receptor binding differ, although the reasons for this remain unclear. Using NL63 pseudotyping and soluble S binding to ACE-2-expressing mammalian cells, Hofmann *et al.* (2005) showed that while ACE-2 leads to efficient NL63 S-mediated entry, the binding of NL63 S was apparently lower than that of SARS-CoV S. They later suggested that this may be the consequence of NL63 S protein binding to a different region of ACE-2 (Hofmann *et al.*, 2006). In contrast, following the definition of a minimum receptor-binding domain (RBD) within NL63 S, residues 301–749, Li *et al.* (2007) showed that incubation of a tagged form of the RBD with cell lines expressing a number of natural and synthetic ACE-2 variants indicated that the ACE-2 contact residues critical for binding both SARS-CoV and NL63 S overlap (Li *et al.*, 2007). The binding site for both viruses is distinct from the active site of the enzyme (Guy *et al.*, 2005; Towler *et al.*, 2004), consistent with the fact that treatment of ACE-2-bearing cells with MLN-4760, a potent ACE-2 inhibitor, has no effect on S–RBD interaction or virus entry (Li *et al.*, 2005b). However, while confirming that full-length SARS-CoV S appeared to bind to ACE-2-expressing cells more efficiently than NL63 S, the SARS-CoV RBD was less efficient at competing with NL63-mediated pseudotype entry than competition with SARS-CoV-mediated entry (IC_50_ of ∼200 ng ml^−1^ for NL63 compared with ∼50 ng ml^−1^ for SARS-CoV) (Li *et al.*, 2007). A recent report suggests that the minimum NL63 RBD lies between residues 476 and 616 and that, expressed alone, the binding affinity with ACE-2 reaches that observed for SARS-CoV S (Lin *et al.*, 2008). Here, using a wholly recombinant system that precludes the involvement of any other cell surface factor present on mammalian cells, such as lectins [e.g. DC-SIGN (Marzi *et al.*, 2004)], we show that the soluble forms of both SARS-CoV and NL63 S protein bind soluble ACE-2 *in vitro* with substantially different affinity. Discrepant binding is maintained but reduced when the S proteins are displayed on the cell surface where avidity may compensate for inherent affinity changes. We confirm that ACE-2 residues shown to be critical for SARS-CoV S binding also abolish NL63 S binding and that the binding of NL63 S does not involve its unique amino-terminal sequence. Differences in the affinity of ACE-2 interaction with the different CoV S proteins are therefore independent of assay format and may underlie the different pathological outcomes of infection.

To investigate CoV S protein binding to ACE-2 in a unified format, we produced soluble and cell-bound versions of the two S proteins through the use of baculovirus expression vectors designed for secretion of proteins as Fc-tagged fusion proteins (Chen *et al.*, 2007) or, separately, displayed on the insect cell surface following tagging with the VSV G protein transmembrane (TM) domain (Chapple & Jones, 2002) (see Supplementary Fig. S1, available in JGV Online). The vectors are isogenic except for the tags employed and lead to the abundant expression of the target glycoproteins in the cells and supernatant for both the SARS-CoV and NL63 S proteins and subdomains thereof (Fig. 1). In addition, we produced a secreted form of ACE-2 fused to green fluorescent protein (GFP) to provide a ligand with an alternate tag for the detection of ligand binding. In order to validate the interaction assays used, we produced two variants of the SARS-CoV S1 domain with alanine substitutions at arginine 426 (R426A) or asparagine 473 (N473A), known to reduce the affinity of SARS-CoV S1 interaction with ACE-2 (Chakraborti *et al.*, 2005). In addition, we introduced two alanine substitutions in the ACE-2 sequence at tyrosine 41 (Y41A) and lysine 353 (K353A), both of which have been mapped as important for the interaction of ACE-2 with SARS-CoV S (Li *et al.*, 2005b). With these differentially tagged and mutated potential ligands, we assessed the interaction of the CoV S proteins with ACE-2 by ELISA, pull-down and flow cytometry assays.

Preliminary ELISAs capturing ACE-2-GFP to the plate, followed by incubation with the various Fc-tagged forms of S protein and detection with an anti-human Ig conjugate showed detectable interaction with SARS-CoV S and S1, but no interaction with either S1 R426A or N473A as expected (data not shown). Ligand binding was equally demonstrable in the alternate format of S capture followed by ACE-2–GFP and an anti-GFP probe. However, at equivalent concentrations of S protein, no interaction with NL63 S or any of its derivatives was detected in either assay format, suggesting an affinity of interaction for NL63 S with ACE-2 in solution that is substantially lower than that of SARS-CoV S. Supernatants containing Fc-tagged CoV S proteins were concentrated by spin dialysis, the levels of S protein present were normalized by quantitative Western blot and equivalent amounts of Fc-tagged S proteins were incubated with ACE-2–GFP in solution for 1 h at 21 °C. Following pull-down of the Fc components with protein A conjugated to Sepharose beads, the recovered contents were resolved by SDS-PAGE and the presence of CoV S proteins and GFP-tagged ACE-2 was detected by Western blot using anti-human Ig and anti-GFP antibodies, respectively. We observed that while SARS-CoV S and S1 pulled down ACE-2 effectively, NL63 S proteins pulled down between 10- and 100-fold less ACE-2 on a weight-for-weight basis (Fig. 2a[Fig f2]). ACE-2 interaction was apparent for NL63 S, NL63 S_15–739_ and NL63 S_196–739_ but not for NL63 S_15–195_ (subscript numbers indicate the residues contained within each fragment) confirming that the unique 180 residue amino terminus of NL63 does not bind ACE-2 (Hofmann *et al.*, 2006; Li *et al.*, 2007) (Fig. 2). Using SARS-CoV S1 and the minimum NL63 binding construct, NL63 S_196–739_, we tested the relative pull-down of ACE-2 with substitutions at residues 41 and 353. SARS-CoV S1 failed to interact significantly with ACE-2 Y41A (∼2 %), it had reduced but demonstrable binding with K353A (∼10 %) and did not pull down the double mutant (Fig. 2b[Fig f2]). NL63 S_196–739_ also failed to effectively pull down ACE-2 Y41A and did not pull down either ACE-2 K353A or the double mutant (Y41A+K353A) (Fig. 2b[Fig f2]). Together these data show that NL63 S has a lower innate affinity for ACE-2 when compared with SARS-CoV S, and confirm the results of Li *et al.* (2007) which show that NL63 S and SARS-CoV S bind an overlapping ACE-2 sequence. However, this study demonstrated that the role of ACE-2 residue 353 was noticeably different. Substitution of the resident lysine for alanine reduced, but did not abolish, SARS-CoV S binding, while it effectively abolished binding by NL63 S. In addition, as the end points in our constructs differed somewhat from those published by Li *et al.* (2007), our data reduced the carboxy-terminal boundary of the NL63 S RBD in an analogous Fc-fusion protein configuration from residue 749 to 739; however, this boundary is not reduced as far as residue 616 as recently described by Lin *et al.* (2008). An affinity of interaction with ACE-2 by NL63 S of 10–100-fold less than that of SARS-CoV S would explain our inability to obtain a sound *K*_d_ for the interaction by surface plasmon resonance. The SARS-CoV S1 affinity with ACE-2 was 8.71×10^−8^ M (Supplementary Fig. S2, available in JGV Online), which is similar to published values (Li *et al.*, 2005b). However, the same assay format failed to record an accurate *K*_d_ for the interaction of NL63 S with ACE-2.

To assess whether the low affinity of NL63 for ACE-2 might be partly compensated for by avidity effects of the S proteins clustering on the viral surface, we used the same S protein domains expressed as VSV G TM fusions on the insect cell surface to examine binding with soluble ACE-2. Using flow cytometry with insect cells expressing S proteins at 2 days post-infection (p.i.), when expression levels were saturating (Fig. 1), incubation of infected cells with an excess of ACE-2–GFP resulted in a two- to threefold difference in ACE-2 binding between SARS-CoV and NL63 S1 (Fig. 3a[Fig f3]). When the range of NL63 S protein fragments was assessed, ACE-2 binding was observed for all the S derivatives, with the exception of the unique amino-terminal domain S_15–195_ (Fig. 3b[Fig f3]), which is in agreement with the solution phase pull-down assays. When SARS-CoV S1- and NL63 S_19–739_-decorated insect cells were incubated with ACE-2 mutants Y41A and K353A, reduced binding was observed for both proteins; the relative reduction in binding parallelled that seen in the pull-down assays. Neither S protein bound the Y41A mutant significantly; there was low residual binding by the K353A mutant to displayed SARS-CoV S1 but no binding to displayed NL63 protein. We conclude that multivalent presentation of the S protein on the cell surface retains the differential binding affinities shown by solution-phase-binding for SARS-CoV and NL63 S proteins, that is, that SARS-CoV S protein has a significantly higher affinity for ACE-2 than NL63 S protein, but that multivalency partly reduces the factor of difference. Densitometry of the pull-downs revealed a >10-fold difference in solution (Fig. 2), while peak fluorescence showed an approximately threefold difference at the cell surface (Fig. 3). We speculate that while NL63 can use ACE-2 as a receptor for virus entry almost as effectively as SARS-CoV, the consequence of binding on events downstream of ACE-2 binding may be different. This is consistent with the relative levels of cell entry exhibited by viruses pseudotyped by SARS-CoV S and NL63 S (SARS-CoV>NL63) (Hofmann *et al.*, 2006); however, a soluble purified RBD of SARS-CoV S was a relatively weak competitor for NL63 S-mediated pseudotype virus entry (Li *et al.*, 2007). In addition, we confirmed that ACE-2 residues key to SARS-CoV S binding are also involved in NL63 binding but that the contribution of individual residues, exemplified here by K353A, may differ. This will relate to the molecular contact between S protein and the receptor, which has been described for SARS-CoV but remains unknown in NL63 (Li *et al.*, 2005a), despite the identification of residues critical for contact (Lin *et al.*, 2008). It remains to be determined exactly what difference in ACE-2 signalling, if any, follows SARS-CoV and NL63 S protein binding and whether this relates to the pathology of infection.

## Supplementary Material

[Supplementary Figures]

## Figures and Tables

**Fig. 1. f1:**
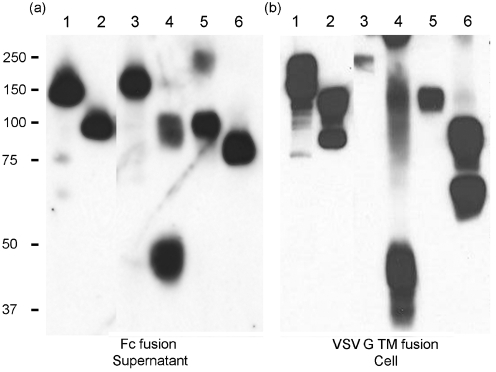
Expression of SARS-CoV and NL63 S proteins using recombinant baculoviruses. Western blots of expression at 2 days p.i. with viruses encoding proteins either as Fc fusion proteins (a) or following fusion to the VSV G TM domain (b). In (a), supernatant was probed with anti-human Ig; in (b), the cell pellet was probed with anti-VSV G protein. Lanes: 1, SARS-CoV S; 2, SARS-CoV S1 (residues 19–713); 3, NL63 S; 4, NL63 S_15–195_; 5, NL63 S_15–739_; 6, NL63 S_196–739_. Molecular mass markers are indicated (kDa).

**Fig. 2. f2:**
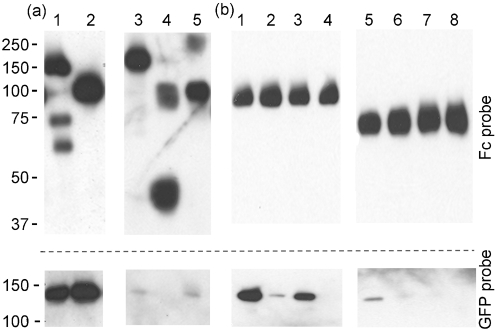
Pull-downs of the various Fc-tagged S proteins after incubation with ACE-2–GFP or ACE-2 mutants. Following SDS-PAGE, the blots were probed with anti-human Ig to detect the S proteins (Fc, upper panels) or anti-GFP to detect ACE-2 (GFP, lower panels). (a) The ligand was ACE-2 wild-type. Samples in lanes 1–5 are SARS-CoV S, SARS-CoV S1 (residues 19–713), NL63 S, NL63 S_15–195_, NL63 S_15–739_, respectively. (b) The ligand was wild-type ACE-2 (lanes 1 and 5), ACE-2 Y41A (lanes 2 and 6), ACE-2 K353A (lanes 3 and 7) or ACE-2 Y41A+K353A (lanes 4 and 8). Samples are pull-downs with SARS-CoV S1 (lanes 1–4) and NL63 S_196–739_ (lanes 5–8). Molecular mass markers are indicated (kDa).

**Fig. 3. f3:**
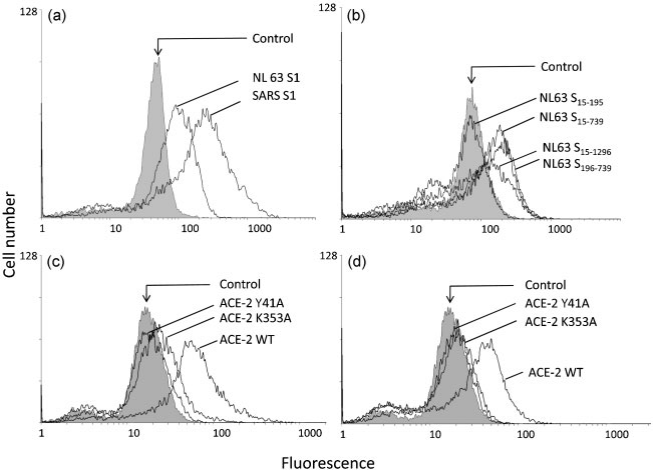
The interaction of CoV S proteins with ACE-2 measured by flow cytometry. Insect cells infected with recombinant baculoviruses expressing the VSV G-tagged form of S protein were used at 2 days p.i. and incubated with either ACE-2 or ACE-2 mutants. (a) Direct comparison between SARS-CoV S1 and the NL63 equivalent incubated with wild-type ACE-2. (b) Comparison between the different fragments of NL63 S using wild-type ACE-2. (c, d) Comparisons between SARS-CoV S1 (c) and NL63 S1 (d) incubated with ACE-2 mutants Y41A and K353A. Note the residual shift by SARS-CoV S1 to K353A, which is not apparent for NL63.
