# Supplementary material for: Interaction of severe acute respiratory syndrome-coronavirus and NL63 coronavirus spike proteins with angiotensin converting enzyme-2
Source: J Gen Virol. 2008 Nov;89(Pt 11):2741–5. doi: 10.1099/vir.0.2008/003962-0 (PMC2886958; doi:10.1099/vir.0.2008/003962-0)
Supplement: [Supplementary Figures] [file supp_89_11_2741__index.html]

 Interaction of severe acute respiratory syndrome-coronavirus and NL63 coronavirus spike proteins with angiotensin converting enzyme-2 -- Mathewson et al. 89 (11): 2741 Data Supplement - Supplementary Figures -- Journal of General Virology

### Interaction of SARS-CoV and NL63 coronavirus spike proteins with ACE-2, by A. C. Mathewson, A. Bishop, Y. Yao, F. Kemp, J. Ren, H. Chen, X. Xu, B. Berkhout, L. van der Hoek and I. M. Jones
